# Diversity, Antimicrobial Activity, and Antibiotic Susceptibility Pattern of Endophytic Bacteria Sourced From *Cordia dichotoma* L

**DOI:** 10.3389/fmicb.2022.879386

**Published:** 2022-05-13

**Authors:** Mahima Sharma, Sharada Mallubhotla

**Affiliations:** Tissue Culture Laboratory, School of Biotechnology, Shri Mata Vaishno Devi University, Katra, India

**Keywords:** bacterial endophytes, Lasura, antimicrobial activity, antibiotic susceptibility, surface sterilization

## Abstract

Endophytic bacteria isolated from medicinal plants are crucial for the production of antimicrobial agents since they are capable of possessing bioactive compounds with diverse structures and activities. *Cordia dichotoma*, a plant of medicinal importance native to the Jammu region of India, was selected for the isolation and characterization of culturable endophytic bacteria and evaluation of their antimicrobial activities. Standardized surface sterilization methods were employed to isolate thirty-three phenotypically distinguishable endophytic bacteria from the root, stem, and leaf parts of the plant. Shannon Wiener diversity index clearly divulged diverse endophytes in roots (0.85), stem (0.61), and leaf (0.54) tissues. Physio-biochemical features of the isolates differentiated the distinct variations in their carbohydrate utilization profile and NaCl tolerance. The endophytes produced an array of enzymes, namely, catalase, oxidase, amylase, cellulase, nitrate reductase, and lipase. The bacterial isolates belong to the genera *Bacillus, Pseudomonas, Paenibacillus, Acidomonas, Streptococcus, Ralstonia, Micrococcus, Staphylococcus*, and *Alcalignes* predominantly. However, the antibiotic susceptibility pattern indicated that the isolates were mostly sensitive to erythromycin and streptomycin, while they were resistant to rifampicin, amoxicillin, and bacitracin. Interestingly, majority of the bacterial endophytes of *C. dichotoma* showed antimicrobial activity against *Bacillus subtilis* followed by *Klebsiella pneumoniae*. The 16S rRNA sequence of *Bacillus thuringiensis* has been deposited in the NCBI GenBank database under accession number OM320575. The major compounds of the crude extract derived from endophytic *B. thuringiensis* OM320575, according to the metabolic profile examination by GC-MS, are dibutyl phthalate, eicosane, tetrapentacontane, heneicosane, and hexadecane, which possessed antibacterial activities. In conclusion, results indicated the potential of *C. dichotoma* to host a plethora of bacterial endophytes that produce therapeutic bioactive metabolites.

## Introduction

Global health problems due to the evolution of defiance to accessible antibiotics by pathogenic fungi and bacteria, the inefficacy of present antifungal and antibacterial agents to various fungal and bacterial infections, and the emergence of life-threatening viruses require a critical need to search for novel and effective antimicrobial agents (Monowar et al., 2018). Numerous factors are responsible for antibiotic resistance, such as poor hygienic conditions, inappropriate use of antibiotics, late diagnosis of infections, and immunocompromised patients (Subramani et al., 2017). Medicinal plants are a source of diverse compounds that can be used for the treatment of human illnesses (Daniel et al., 2018), and due to their biological friendly nature and bioactive compounds, they are used as pharmaceutical agents (Waheed et al., 2013). Recently, natural compounds obtained from plants, fungi, and bacteria have been sourced to treat multidrug-resistant contagious pathogens singly or in combination with antibiotics (Mai et al., 2019). The use of natural antimicrobial agents is also preferred because they form protein-protein bonds during the interaction, and thus, microbes infrequently develop resistance against them (Nisa et al., 2020). Recently, search for novel curative agents has been focused toward endophytes from plant hosts because of their numerous applications of novel and interesting bioactive compounds.

Endophytes are microbes (bacteria and fungi) that colonize inner healthy plant tissues without causing them any pathogenicity (Wilson, 1995). Nearly all plants are thought to associate with endophytic microbes, yet some plant species have never been entirely studied for endophytes (Mengistu, 2020). Endophytic microbes are considered natural reservoirs due to their ability to produce myriad bioactive compounds (Gouda et al., 2016). Valuable bioactive metabolites, such as alkaloids, steroids, terpenoids, lactones, quinines, lignans, and phenols, have been isolated from endophytic fungi and bacteria (Deshmukh et al., 2015). Endophytic bacteria from medicinal plants have also been considered for their antimicrobial activities (Wang et al., 2019; Xu et al., 2020). Bacterial endophytes could also produce metabolites alike or with additional prominent activity than that of their respective hosts (Venieraki et al., 2017).

Bacterial endophytes are thought to have a symbiotic relationship with plants. In this mutualistic relationship, plants give shelter and nutrients to the endophytes (Liarzi et al., 2016), while host plants are protected from herbivores and pathogens (Qin et al., 2017). Additionally, by producing phytohormones, endophytic bacteria also promote plant growth, thus enhancing their resistance to various abiotic stresses, i.e., heavy metal toxicity and salinity (Khan et al., 2014), and they can be used in agriculture, industry, and medicine (Ryan et al., 2008). Studies on the isolation of bioactive products from bacterial endophytes can help in the discovery of several new compounds that can also be developed as antimicrobial agents to manage antibiotic resistance.

*Cordia dichotoma*, commonly known as Lasura, is the main plant with ethnobotanical importance belonging to the family Boraginaceae and is widespread in different parts of India. Its bark paste is useful to treat stomach disorders and to relieve chest pain. Traditionally, all parts of the plant are used to treat various illnesses, such as wound healing, antiulcer, antihelmintic, urinary infections, analgesic, antitumor, antifertility, antimicrobial, dysentery, dyspepsia, cough, and jaundice (Ragasa et al., 2015; Kumari et al., 2016). Due to its diverse therapeutic uses, this plant was selected to study its related bacterial endophytes and to screen the strains for their antimicrobial potential against a panel of clinically serious human pathogens. This plant has not been estimated for the isolation of bacterial endophytes and their activities. In this study, we estimated the diversity and antibacterial potential of bacterial endophytes colonizing *C. dichotoma*.

## Materials and Methods

### Collection of Plant Sample

Healthy plant parts, i.e., root, stem, and leaf, were collected from three individual plants of *C. dichotoma* growing in the Herbal Garden of Shri Mata Vaishno Devi University, Katra, Jammu and Kashmir (32.9418°N and 74.9541°E, elevation 754 m), India, in sterile polythene bags and brought immediately to the laboratory and processed to reduce the risk of contamination. The specimen was deposited at the Department of Botany, University of Jammu, with accession number 16613.

### Isolation of Endophytic Bacteria

#### Surface Sterilization

The collected plant samples (i.e., root, stem, and leaf) were washed under running tap water to remove the dust and debris present on the surface, followed by washing in distilled water. They were carefully excised and exposed to four different reported surface sterilization methods. In Method I, samples were surface sterilized with 70% ethanol for 8 min (Tian et al., 2007). In Method II, samples were immersed in 70% ethanol for 2 min, then washed with 1% sodium hypochlorite for 3 min, and finally rinsed with 70% ethanol for 1 min (Khanam and Chandra, 2017). In Method III, samples were surface sterilized with 0.1% mercuric chloride for 2 min, 70% ethanol for 1 min, and 2% sodium hypochlorite for 5 min (Baldan et al., 2014). In Method IV, samples were immersed in Tween 20 (0.1% in 100 ml sterile distilled water), then washed with 4% sodium hypochlorite for 10 min, and finally rinsed with 70% ethanol for 3 min (Ramalashmi et al., 2018). After each step, all plant samples were washed with sterile distilled water, and each method was carried out in triplicates.

### Sterility Test

Plant samples were washed in sterile distilled water at every step of the surface sterilization process. To assess the effectiveness of the surface sterilization method, a sterility check was performed for every sample. For this surface sterilization, plant samples were placed on nutrient agar, incubated at 37°C for 48 h, and checked for feasible microbial growth. Plant samples were used for further experimentation only if no growth was observed (Hallmann et al., 2006).

### Isolation, Purification, and Preservation

The surface-sterilized plant segments were cut into approximately 6 mm diameter disks under aseptic conditions and placed on nutrient agar plates, and they were incubated for 48 h at 37°C in an inverted position. After 48 h, the plates were observed for bacterial growth surrounding the root, stem, and leaf sections. Endophytic bacteria emerging from the surface of plant segments were collected using an inoculation needle and further subcultured to obtain pure colonies. Single colonies acquired were streaked on fresh nutrient agar plates for further purification. These pure colonies were preserved in 50% glycerol stock and used for further experimental procedures.

### Characterization of Bacterial Isolates

All selected bacterial isolates were identified through morphological characteristics, such as colony color, margin, consistency, and texture, and microscopic characterization, such as gram staining, endospore staining, and motility. The biochemical and physiological tests like indole utilization, methyl red, and sugar utilization were performed according to standard procedures (Pielou, 1975; Smibert and Krieg, 1994).

### Enzyme Activity

The endophytic bacterial strains were screened for the presence of several enzyme activities, such as catalase, oxidase, amylase, cellulase, and lipase. The monitoring was done by streaking the selected endophytic bacteria on culture agar media with the substrate (Table 1 in Supplementary Material). For lipase, cellulase, and amylase activities, standard substrates like tributyrin, carboxymethyl cellulose, and starch were used, respectively. Enzyme activity was observed by flooding the plates with Congo red for cellulase and iodine reagent for amylase, and the presence of clear zones was confirmed for lipase. Catalase and oxidase activities of the bacterial isolates were determined by adding hydrogen peroxide (H_2_O_2_) and oxidase reagent (1% tetramethyl-p-phenylenediamine dihydrochloride), respectively, on the smear of freshly grown endophytic bacterial cultures in nutrient agar plates. The presence of oxygen bubbles and purple color, respectively, was considered as positive.

### Salt Tolerance

To examine the effect of sodium chloride on the growth of endophytic bacterial isolates, a nutrient agar medium was prepared by adding different concentrations of NaCl (0–12%). Endophytic bacterial strains were inoculated into the plates and incubated at 37°C for 6 days, and observations were recorded every 24 h.

### Antibacterial Evaluation

All endophytic bacterial strains were grown in nutrient broth medium for 5 days at 37°C, and antimicrobial activity was determined by agar diffusion method against six pathogenic bacteria, namely, *Staphylococcus aureus* MTCC 737*, Escherichia coli* MTCC 1687, *Bacillus subtilis* MTCC 1789*, Pseudomonas aeruginosa* MTCC 1688*, Klebsiella pneumonia* MTCC 432, and *Salmonella typhi* MTCC 733. Each endophytic bacterial culture (10 ml) was centrifuged at 1,000 rpm for 10 min and the supernatant was used to determine antibacterial activity. Overnight grown cultures of the test organisms were spread by sterile cotton swabs onto the surface of the Muller Hinton agar plates. Wells (6 mm) were made and 100 μl of endophytic bacterial strain was added into it; an equal volume of sterile nutrient broth instead of bacterial endophyte was used as a negative control, and ampicillin was used as a positive control. All plates were wrapped with parafilm and incubated for 24 h at 37°C and observed for the zone of inhibition of pathogenic bacteria. Antibacterial activity was evaluated by measuring the diameter of the clear zone of inhibition (Mohamad et al., 2018).

### Antibiotic Susceptibility Assay

Antibiotic susceptibility test of endophytic bacterial strains was performed by adopting the Kirby-Bauer disk diffusion method (Bauer, 1966). Overnight nutrient broth culture of endophytic bacteria was prepared, and all the endophytic bacterial isolates were inoculated into Muller Hinton agar plates using a sterile cotton swab. Standard antibiotic disks, such as ampicillin (10 μg/disk), streptomycin (30 μg/disk), neomycin (30 μg/disk), chloramphenicol (30 μg/disk), bacitracin (10 U/disk), rifampicin (5 μg/disk), erythromycin (15 μg/disk), kanamycin (30 μg/disk), amoxycillin (30 μg/disk), and tetracycline (30 μg/disk), were placed on Muller Hinton agar plates and incubated 37°C for 24 h. After incubation, the antibiotic susceptibility pattern was determined by measuring the inhibition zone. Based on the diameter of the zone of inhibition recorded to the nearest mm, the organisms were designated as sensitive, intermediate, and resistant following DIFCO Manual 10th edition (1984).

### Molecular Identification of Endophytic Bacterial Isolate

Bacterial isolates with high antibacterial activity were chosen for molecular identification using 16S rDNA sequence analysis. A loopful of freshly grown bacterial cells was dissolved in tubes with 500 μl of Tris-EDTA for DNA extraction. Then, 500 μl of phenol: chloroform: isoamyl alcohol (25:24:1) was added. This mixture was vortexed and centrifuged at 14,000 *g* for 5 min. The upper aqueous phase was transferred to a fresh tube, and 800 μl of chilled isopropanol was added. The samples were centrifuged again for 5 min at 14,000 *g*, and the supernatant was discarded. The pellets were dried at room temperature overnight before being eluted in 70 μl of TE buffer. A NanoDrop spectrophotometer was used to measure the absorbance at 260 nm to determine the concentration of genomic DNA. The primers 27F (5′-AGAGTTTGATCCTGGCTCAG-3′) and 1492R (5′-GGTTACCTTGTTACGACTT-3′) were used to amplify the 16S rRNA gene. Total DNA (50–500 ng) was added to the PCR mix (30 μl), which contained 24 μl of DreamTaq PCR master mixture, 1 μl of 10 nm each primer, 2 μl of DNA, and 7.5 μl of 10X buffer in a total volume of 30 μl. This reaction was performed under the following conditions: one cycle of 94°C for 5 min, followed by 35 cycles of 94°C for 1 min, 55°C for 1 min, 72°C for 1 min, and a final extension of 10 min at 72°C. The amplified DNA products were separated and visualized under UV light using agarose gel electrophoresis. The amplicons were purified using the Genei Pure^TM^ quick PCR purification kit and quantified using a spectrophotometer at 260 nm. Biologia Research India sequenced the purified partial 16S rDNA amplicons. The sequences were assembled, edited, and aligned in MEGA11 before comparison with those in the GenBank database with the Basic Local Alignment Search Tool (https://blast.ncbi.nlm.nih.gov/Blast.cgi) to determine the sequence homology with closely related organisms. In this study, the microorganisms with the highest level of identity (100%) were chosen as the closest match, and isolated bacteria were classified to the species level based on the information of the closest microbes.

### Estimation of Chemical Components Using GC-MS Analysis

To analyze the various volatile bioactive constituents, an ethyl acetate extract of SMC212 was subjected to gas chromatography-mass spectrometry (GC-MS). The Indian Institute of Technology, Jammu, conducted the GC-MS analysis. SHIMADZU, QP2010 PLUS was used for GC-MS analysis. The injecting temperature was set to 250°C, the column temperature was set to 50°C, the pressure was set to 29.7 kPa, and the column flow rate was set to 0.72 ml/min. The sample's total running time was 40 min. The phytochemical compounds in ethyl acetate extract were identified based on retention time by matching MS with available standards using the Willey and NIST libraries. The constituents' names, molecular formulas, and molecular weights were determined.

### Statistical Analysis

Colonization rate was determined as the total number of plant segments colonized by bacteria divided by the total number of incubated samples. Isolation rate was calculated as the number of bacterial strains obtained from plant segments divided by the total number of incubated segments. The Shannon Weiner diversity index (*H* ^/^) was calculated as:


(1) $$\begin{array}{r} H^{/}=-\Sigma \text{Pi}Xln\text{Pi}, \end{array}$$

where *Pi* is the relative abundance of species and *i* contributes to the total number of species (Di Bitetti, 2000). All of the experiments were carried out in triplicate, and the data reported are the averages of the results. The SPSS-22 statistical software (SPSS, Inc., Chicago, IL, USA) was used to calculate the means and standard deviation.

## Results

### Efficiency of Surface Sterilization Methods

**Figure 1:**
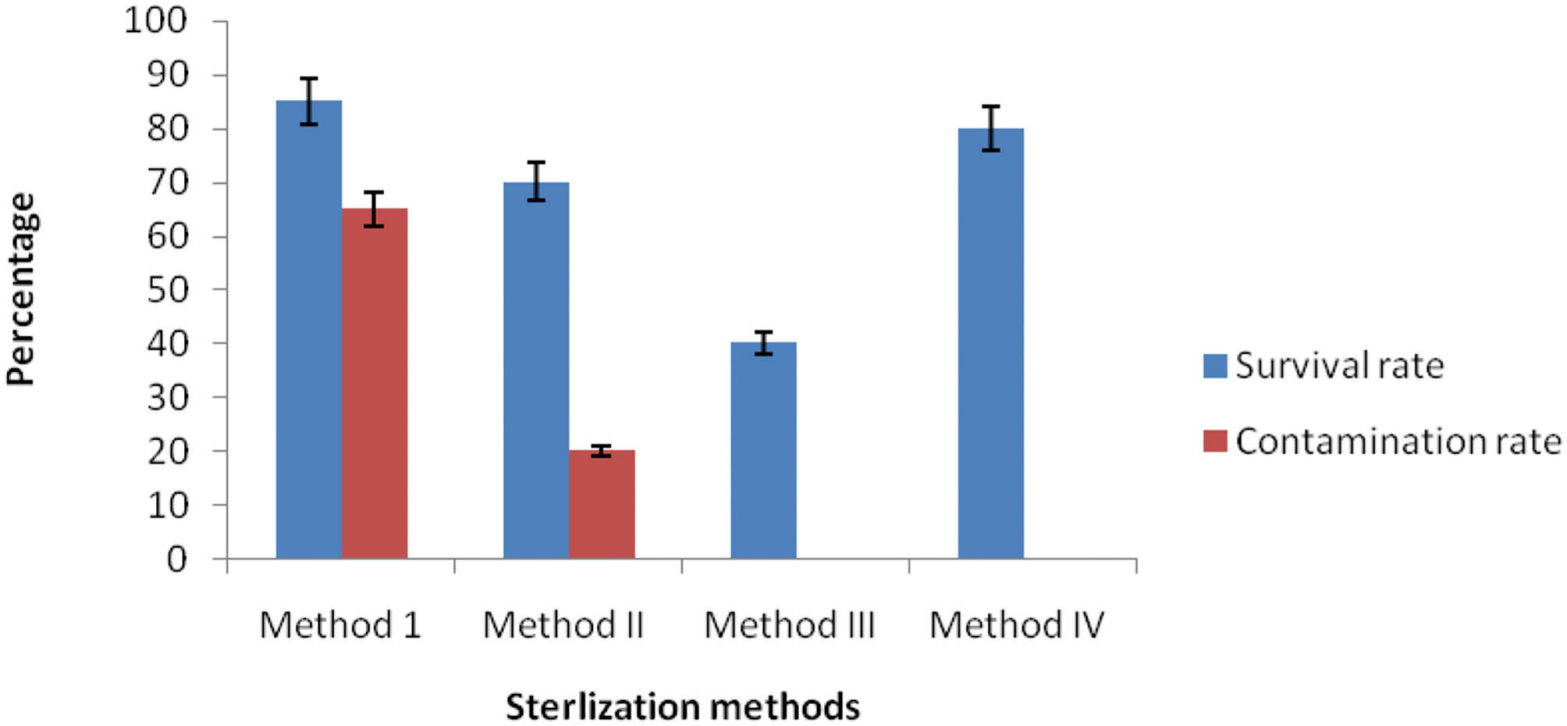
Efficiency of the surface sterilization methods of *C. dichotoma*.

To get pure endophytes from the inner plant tissue*s* of *C. dichotoma*, epiphytic microorganisms and other contamination must be removed or killed through the surface sterilization method. For this, plant samples (i.e., root, stem, and leaf) were treated separately by a different amalgam of chemical disinfectants. Method I (70% ethanol) was not found productive individually as a high percentage of contamination was noticed along with the growth of endophytes. While in Methods II, III, and IV, plant samples were treated with different mixtures and duration of sodium hypochlorite, ethanol, and mercuric chloride to attain a sufficient result. In Method III, mercuric chloride was successful in removing the contamination, yet the survival percentage of endophytic bacteria declined. Our result shows that only Method IV (0.1% Tween 20, 4% sodium hypochlorite for 10 min, and 70% ethanol for 3 min) was found effective for surface sterilization of *C. dichotoma* plant tissues, with no contamination and a high percentage of survival rate. Reports are indicative of surface sterilization methods used in dye-yielding plants for the isolation of endophytic bacteria with 70% ethanol for 6–8 min and 0.1% mercuric chloride for 5–10 min showing promising results (Khanam and Chandra, 2017). It was found that surface sterilization by 2% sodium hypochlorite and 0.1% Tween 20 for 3 min followed by ethanol 70% for 1 min was well-suited for the isolation of endophytic microorganisms from *Acalypha indica* (Ramalashmi et al., 2018). The results for optimization of surface sterilization are shown in Figure 1.

### Isolation of Endophytic Bacteria

Segments of surface-sterilized root, stem, and leaf of *C. dichotoma* incubated on nutrient agar plates showed growth of morphologically distinct bacterial colonies surrounding the plant segments after 48–96 h. To avoid the collection of repetitive strains, a total of 33 endophytic bacteria were isolated in pure form from 170 segments (i.e., 62 root, 54 stem, and 54 leaf segments) of *C. dichotoma* of which 16, 9, and 8 isolates were obtained from root, stem, and leaf segments, respectively (Table 1; Figure 2 in Supplementary Material). Colonization frequency calculated in percentage was highest in leaf samples (37.03) but gradually decreased in stem (35.18) and root (32.25) samples, while the isolation rate was lower in leaf (0.14) as compared to stem (0.16) and root (0.25) samples. The Shannon Wiener diversity index showed that roots (0.85) of *C. dichotoma* harbor diverse types of endophytic bacteria as compared with stem (0.61) and leaf (0.54) which are in line with the available reports for Chinese medicinal plants *Berberis poiretii, Eucommia ulmoides*, and *Rhus potanini* showing a high colonization frequency (47–63%) and isolation rates (0.7–0.9) of endophytic fungi (Sun et al., 2008).

**Table 1:** Diversity of endophytic bacterial isolates in root, stem, and leaf tissues of *C. dichotoma*.

**Parameters**	**Plant parts**			
	**Root**	**Stem**	**Leaf**	**Total**
Number of samples	62	54	54	170
Number of sample yielding isolates	20	19	20	59
Number of isolates	16	9	8	33
Colonization frequency, %	32.25	35.18	37.03	34.70
Isolation rate	0.25	0.16	0.14	0.19
Shannon wiener diversity index	0.85	0.61	0.54	0.68

### Characterization of Endophytic Bacteria

The bacterial endophytes of *C. dichotoma* were characterized based on morphological (Table 3 in Supplementary Material), microscopical, and biochemical features (Table 2); evaluation of enzymatic activity; and carbohydrate utilization (Table 3). Cultural characteristics of all the isolated endophytic bacteria showed that out of 33 isolates, 18 pigmented and 15 non-pigmented organisms were identified. Microscopic characteristics of all endophytic bacteria showed that out of 33 isolates, 18 were Gram-positive (5 cocci and 13 rods) and 15 were Gram-negative rods. Filamentous forms were not observed in either root, stem or leaf samples. Twelve isolates out of 33 were motile, and 8 isolates showed endospore formation, and they were suspected as *Bacillus* species. Enzyme profile of endophytic bacterial isolates showed that all endophytic bacteria produced oxidase, and 87% of them produced catalase. The isolates also showed lipase (69%), amylase (78%), cellulase (87%), nitrate reductase (39%), methyl red (30%), and indole (39%) production. The isolated endophytes were also screened for their ability to utilize carbohydrates, i.e., dextrose, fructose, sucrose, maltose, and lactose, in phenol red agar medium supplemented with 1% sugar. Dextrose was found to be the best-utilized carbon source by most of the bacterial isolates; bacterial isolates were moderate in fermenting fructose and sucrose, while very few isolates were fermenting maltose (6) and lactose (7).

**Table 2:** Microscopical and biochemical characterization of endophytic bacteria.

**Plant**	**Bacterial**	**Gram**	**Shape**	**Motility**	**Endospore**	**Indole**	**MR**	**Nitrate**	**H_**2**_S**
**part**	**code**	**staining**				**production**		**reductase**	
Root	SMC201	G–ve	Rod	–	–	–	–	–	–
	SMC202	G–ve	Rod	–	–	–	+	–	–
	SMC203	G+ve	Cocci	–	–	–	–	+	–
	SMC204	G–ve	Rod	+	–	+	+	+	–
	SMC205	G–ve	Rod	+	–	–	+	–	+
	SMC206	G+ve	Rod	–	+	–	–	+	+
	SMC207	G–ve	Rod	–	–	+	–	–	–
	SMC208	G+ve	Cocci	–	–	+	+	–	–
	SMC209	G+ve	Rod	+	+	–	–	–	+
	SMC210	G+ve	Rod	–	–	+	–	+	–
	SMC211	G–ve	Rod	–	–	–	–	–	–
	SMC212	G+ve	Rod	+	+	–	–	+	–
	SMC213	G–ve	Rod	+	–	+	–	–	–
	SMC214	G+ve	Rod	+	+	–	–	+	+
	SMC215	G+ve	Rod	–	–	–	+	–	–
	SMC216	G–ve	Rod	–	–	–	–	–	–
Stem	SMC101	G+ve	Rod	+	–	+	–	–	–
	SMC102	G–ve	Rod	+	–	–	+	+	–
	SMC103	G–ve	Rod	–	–	+	–	+	–
	SMC104	G+ve	Rod	–	+	+	–	–	–
	SMC105	G+ve	Cocci	–	–	–	+	–	+
	SMC106	G+ve	Rod	+	–	+	–	+	–
	SMC107	G–ve	Rod	–	–	–	–	–	–
	SMC108	G–ve	Rod	–	–	–	–	–	+
	SMC109	G+ve	Rod	–	+	+	+	+	–
Leaf	SMC301	G–ve	Rod	+	–	+	–	–	–
	SMC302	G+ve	Rod	+	–	–	–	+	+
	SMC303	G+ve	Rod	–	+	–	–	–	+
	SMC304	G+ve	Cocci	–	–	–	+	–	–
	SMC305	G+ve	Cocci	–	–	+	–	+	–
	SMC306	G–ve	Rod	–	–	+	–	+	–
	SMC307	G–ve	Rod	+	–	–	+	–	+
	SMC308	G+ve	Rod	–	+	–	–	–	–

*+ indicates positive; – indicates negative*.

*G–ve indicates Gram-negative bacteria; G+ve indicates Gram-positive bacteria*.

**Table 3:** Evaluation of enzymatic activity and carbohydrate utilization of endophytic bacteria isolated from root, stem, and leaf tissues of *C. dichotoma*.

**Plant part**	**Bacterial code**	**Enzyme profile**					**Carbohydrate utilization**				
		**Catalase**	**Oxidase**	**Amylase**	**Lipase**	**Cellulase**	**Dextrose**	**Fructose**	**Sucrose**	**Lactose**	**Maltose**
Root	SMC201	+	+	+	+	+	+	–	+	–	–
	SMC202	+	+	+	+	+	+	+	–	–	–
	SMC203	+	+	+	+	+	+	+	–	–	–
	SMC204	+	+	–	+	+	+	+	–	–	–
	SMC205	+	+	+	+	+	+	+	–	–	–
	SMC206	+	+	+	+	+	+	–	+	–	–
	SMC207	+	+	+	+	+	+	–	+	–	–
	SMC208	–	+	+	–	+	–	–	–	+	+
	SMC209	–	+	–	–	–	+	+	–	–	–
	SMC210	+	+	+	–	+	+	+	–	–	–
	SMC211	+	+	+	+	+	+	+	+	–	–
	SMC212	+	+	+	+	+	+	+	+	–	–
	SMC213	+	+	+	+	+	–	–	+	+	+
	SMC214	+	+	+	–	+	–	–	+	–	–
	SMC215	+	+	+	–	–	+	+	–	–	–
	SMC216	+	+	+	+	+	+	+	–	+	–
Stem	SMC101	+	+	–	+	+	+	+	–	–	–
	SMC102	+	+	+	+	+	+	–	–	–	+
	SMC103	+	+	–	+	+	–	+	–	–	–
	SMC104	+	+	+	+	+	–	+	–	+	–
	SMC105	+	+	+	–	–	+	–	–	–	+
	SMC106	+	+	–	+	+	+	+	+	–	–
	SMC107	+	+	+	–	+	+	+	+	–	–
	SMC108	+	+	+	+	+	+	–	–	+	–
	SMC109	+	+	+	+	+	–	+	+	–	–
Leaf	SMC301	–	+	+	–	+	+	–	+	–	+
	SMC302	+	+	+	+	+	–	+	–	+	–
	SMC303	+	+	–	+	+	+	+	–	–	–
	SMC304	+	+	+	+	+	+	–	+	–	–
	SMC305	+	+	–	–	+	+	+	–	–	–
	SMC306	+	+	+	–	+	–	+	+	–	–
	SMC307	–	+	+	+	+	+	–	+	–	+
	SMC308	+	+	+	+	–	+	+	–	+	–

*+ indicates positive; – indicates negative*.

Based on the micromorphological and physio-biochemical analysis, the isolated endophytic bacteria were tentatively identified as species of *Bacillus, Pseudomonas, Paenibacillus, Acidomonas, Streptococcus, Ralstonia, Micrococcus, Staphylococcus*, and *Alcalignes*. Similar endophytic bacteria have been reported from medicinal plants like *Azadirachta indica, Gynura procumbens, Tephrosia apolline, Phyllanthus emblica*, and *Vitis vinifera* (Baldan et al., 2014; Khan et al., 2014). However, several authors have reported the presence of endophytic bacteria inside plants belonging to the genera *Pantoea, Bacillus, Microbacterium, Paenibacillus*, and *Sphingomonas* (Rijavec et al., 2007).

### Effect of Sodium Chloride

The growth of isolated endophytic bacteria at different sodium chloride concentrations (Table 4) indicates that all the 33 isolates showed good growth at 0–6% NaCl concentration. Only 22 isolates showed sustained growth at 12% NaCl concentration. Thus, it can be inferred that an increase in NaCl concentration causes a proportional decrease in growth rate.

**Table 4:** Effect of sodium chloride on the growth of isolated endophytic bacteria.

**Plant part**	**Bacterial code**	**0%**	**2%**	**4%**	**6%**	**8%**	**10%**	**12%**
Root	SMC201	+++	+++	+++	+++	++	++	+
	SMC202	+++	+++	+++	+++	+++	++	+
	SMC203	+++	+++	+++	+++	+++	++	+
	SMC204	+++	+++	+++	+++	–	–	–
	SMC205	+++	+++	+++	+++	–	–	–
	SMC206	+++	+++	+++	+++	++	+	+
	SMC207	+++	+++	+++	+++	++	++	+
	SMC208	+++	+++	+++	+++	++	++	+
	SMC209	+++	+++	+++	+++	+	–	–
	SMC210	+++	+++	+++	+++	++	++	+
	SMC211	+++	+++	+++	+++	–	–	–
	SMC212	+++	+++	+++	+++	++	+	+
	SMC213	+++	+++	+++	+++	++	++	+
	SMC214	+++	+++	+++	+++	–	–	–
	SMC215	+++	+++	+++	+++	++	++	+
	SMC216	+++	+++	+++	+++	+++	++	+
Stem	SMC101	+++	+++	+++	+++	–	–	–
	SMC102	+++	+++	+++	+++	++	++	+
	SMC103	+++	+++	+++	+++	++	++	+
	SMC104	+++	+++	+++	+++	++	++	+
	SMC105	+++	+++	+++	+++	++	++	+
	SMC106	+++	+++	+++	+++	++	+	–
	SMC107	+++	+++	+++	+++	++	++	+
	SMC108	+++	+++	+++	+++	–	–	–
	SMC109	+++	+++	+++	+++	+++	++	+
Leaf	SMC301	+++	+++	+++	+++	+++	++	+
	SMC302	+++	+++	+++	+++	++	++	+
	SMC303	+++	+++	+++	+++	–	–	–
	SMC304	+++	+++	+++	+++	++	++	+
	SMC305	+++	+++	+++	+++	++	+	–
	SMC306	+++	+++	+++	+++	++	+	–
	SMC307	+++	+++	+++	+++	++	++	+
	SMC308	+++	+++	+++	+++	++	++	+

*+++ indicates good in growth, ++ indicates moderate in growth, + indicates poor in growth*.

### Antibiotic Susceptibility Pattern

The antibiotic susceptibility pattern of the selected endophytic bacteria was determined against ten different antibiotics by the disk diffusion method, which shows that endophytic bacteria from root, stem, and leaf of *C. dichotoma* were mostly resistant to rifampicin, amoxicillin, and bacitracin, while they were susceptible to erythromycin and streptomycin. Out of 33 isolates, 10 and 7 bacteria showed an intermediate response to neomycin and ampicillin, respectively. Out of 33 isolates, 3 isolates were sensitive to rifampicin, 5 isolates were sensitive to amoxicillin and bacitracin, 8 isolates were sensitive to ampicillin, 14 isolates were sensitive to tetracycline and neomycin, 20 isolates were sensitive to chloramphenicol, 22 isolates were sensitive to kanamycin, 23 isolates were sensitive to erythromycin, and 25 isolates were sensitive to streptomycin. Antibiotic sensitivity patterns of isolated endophytic bacteria with zone size in mm are shown in Table 5.

**Table 5:** Antibiotic susceptibility pattern of endophytic bacteria (zone size in mm).

**Plant** **part**	**Bacterial** **code**	**Antibiotic sensitivity (Inhibition zone in mm)**									
		**Chloramphenicol**	**Streptomycin**	**Ampicillin**	**Neomycin**	**Bacitracin**	**Erythromycin**	**Rifampicin**	**Kanamycin**	**Tetracycline**	**Amoxycillin**
Root	SMC201	10 ± 0.4(R)	25 ± 0.8(S)	10 ± 0.8(R)	15 ± 0.8(I)	10 ± 0.4(R)	15 ± 0.8(I)	0(R)	20 ± 0.8(S)	20 ± 0.8(S)	0(R)
	SMC202	20 ± 1.6(S)	30.4 ± 0.8(S)	15 ± 0.4(I)	18.4 ± 0.2(I)	15 ± 0.8(I)	25.4 ± 0.2(S)	9.5 ± 0.4(R)	24 ± 0.4(S)	22 ± 0.4(S)	0(R)
	SMC203	25 ± 0.8(S)	20.3 ± 0.5(S)	19.7 ± 0.4(S)	19.7 ± 0.4(S)	12.3 ± 0.2(R)	23.9 ± 0.6(S)	10 ± 0.4(R)	1 19.2 ± 0.8(S)	15.7 ± 0.6(I)	10 ± 0.2(R)
	SMC204	17.2 ± 0.6(I)	25 ± 0.8(S)	10.5 ± 0.4(R)	20 ± 0.4(S)	19.5 ± 0.4(S)	25 ± 0.5(S)	14.7 ± 0.4(I)	20 ± 0.3(S)	16.9 ± 0.6(I)	0(R)
	SMC205	15.2 ± 0.6(I)	24.4 ± 0.8(S)	0(R)	23 ± 0.8(S)	0(R)	19.9 ± 0.2(S)	10 ± 0.4(R)	25 ± 0.8(S)	14.9 ± 0.2(I)	0(R)
	SMC206	20 ± 0.8(S)	30 ± 0.8(S)	29.9 ± 0.6(S)	19.9 ± 0.6(S)	0(R)	24.9 ± 0.2(S)	9.9 ± 0.1(R)	23.7 ± 0.6(S)	20.4 ± 0.4(S)	20 ± 0.4(S)
	SMC207	20 ± 0.4(S)	25.4 ± 0.8(S)	15.5 ± 0.7(I)	19.5 ± 0.4(S)	16.7 ± 0.6(I)	20 ± 0.4(S)	0(R)	20.2 ± 1.0(S)	9.9 ± 0.6(R)	0(R)
	SMC208	25.4 ± 2.0(S)	30 ± 0.8(S)	24.7 ± 0.4(S)	21.7 ± 0.6(S)	21 ± 0.8(S)	25 ± 0.8(S)	5 ± 0.8(R)	25.3 ± 0.2(S)	20 ± 0.8(S)	0(R)
	SMC209	19.7 ± 0.8(S)	25.4 ± 0.8(S)	20 ± 0.8(S)	19.5 ± 0.4(S)	17.6 ± 0.4(I)	30.5 ± 0.4(S)	10.2 ± 0.2(R)	25.2 ± 0.1(S)	10 ± 0.4(R)	20 ± 0.6(S)
	SMC210	19.7 ± 0.4(S)	30 ± 0.8(S)	19.2 ± 0.6(S)	25 ± 0.8(S)	9.7 ± 0.4(R)	8.2 ± 0.2(R)	10.2 ± 0.1(R)	19.7 ± 0.4(S)	6 ± 0.8(R)	10 ± 0.4(R)
	SMC211	15 ± 0.8(I)	20 ± 0.4(S)	15.9 ± 0.6(I)	17.5 ± 0.4(I)	9.9 ± 0.2(R)	30.2 ± 0.2(S)	16 ± 0.3(I)	24.9 ± 0.2(S)	14.9 ± 0.6(I)	0(R)
	SMC212	25 ± 0.8(S)	25 ± 0.8(S)	19.9 ± 0.6(S)	30 ± 0.8(S)	25 ± 0.8(S)	20 ± 0.4(S)	14.9 ± 0.6(I)	35 ± 0.8(S)	20.1 ± 0.2(S)	15 ± 0.4(I)
	SMC213	27 ± 0.4(S)	20 ± 0.4(S)	19.9 ± 0.2(S)	25 ± 0.4(S)	23.7 ± 0.6(S)	19.7 ± 0.4(S)	20.2 ± 0.2(S)	16 ± 0.8(I)	19.5 ± 0.4(S)	19 ± 0.8(S)
	SMC214	0(R)	0(R)	20.5 ± 0.4(S)	12 ± 0.8(R)	0(R)	0(R)	0(R)	14.7 ± 0.4(I)	0(R)	0(R)
	SMC215	25.2 ± 0.6(S)	20 ± 0.8(S)	15.7 ± 0.9(I)	24.9 ± 0.2(S)	25 ± 0.8(S)	30.3 ± 0.2(S)	17 ± 0.4(I)	0(R)	10 ± 0.8(R)	0(R)
	SMC216	22 ± 0.8(S)	23.7 ± 0.6(S)	9.9 ± 0.6(R)	17.5 ± 0.4(I)	0(R)	24.7 ± 0.4(S)	0(R)	22 ± 0.4(S)	10 ± 0.3(R)	17 ± 0.8(I)
Stem	SMC101	20.4 ± 1.2(S)	25 ± 1.6(S)	10 ± 0.4(R)	15 ± 0.8(I)	19.2 ± 0.2(I)	30 ± 0.4(S)	0(R)	20 ± 0.4(S)	30 ± 0.8(S)	0(R)
	SMC102	0(R)	9.9 ± 0.6(R)	0(R)	9.9 ± 0.6(R)	17.2 ± 0.2(I)	30.2 ± 0.2(S)	0(R)	19.8 ± 0.2(S)	0(R)	20 ± 1.2(S)
	SMC103	15 ± 0.8(I)	20 ± 0.4(S)	15 ± 0.8(I)	10.5 ± 0.4(R)	0(R)	15 ± 0.8(I)	9.8 ± 0.2(R)	0(R)	10 ± 0.8(R)	0(R)
	SMC104	25.4 ± 0.8(S)	9.7 ± 0.4(R)	0(R)	15.7 ± 0.6(I)	10.2 ± 0.2(R)	9.5 ± 0.4(R)	8.4 ± 0.2(R)	25 ± 0.8(S)	20.1 ± 0.2(S)	0(R)
	SMC105	0(R)	20.7 ± 0.9(S)	0(R)	13.5 ± 0.4(R)	9.5 ± 0.4(R)	27.2 ± 0.2(S)	10 ± 0.4(R)	0(R)	19.7 ± 0.2(S)	10 ± 0.6(R)
	SMC106	9.4 ± 0.9(R)	14 ± 0.8(I)	9 ± 0.8(R)	10 ± 0.8(R)	12.3 ± 0.3(R)	9.9 ± 0.6(R)	5 ± 0.8(R)	9.9 ± 0.6(R)	14.9 ± 0.1(I)	0(R)
	SMC107	20.7 ± 1.3(S)	22.2 ± 1.0(S)	0(R)	20.5 ± 0.4(S)	10 ± 0.4(R)	12 ± 0.4(R)	15.7 ± 0.6(I)	20 ± 0.8(S)	20 ± 0.8(S)	10 ± 0.6(R)
	SMC108	22 ± 0.8(S)	25 ± 0.8(S)	10.5 ± 0.4(R)	24.5 ± 0.4(S)	11 ± 0.8(R)	25 ± 0.8(S)	19.9 ± 0.2(S)	20 ± 0.4(S)	20.2 ± 0.2(S)	0(R)
	SMC109	19 ± 0.8(S)	19.2 ± 0.8(S)	15.9 ± 0.6(I)	15.5 ± 0.4(I)	0(R)	19.5 ± 0.4(S)	0(R)	0(R)	0(R)	0(R)
Leaf	SMC301	4.9 ± 0.2(I)	25 ± 0.8(S)	0(R)	15.9 ± 0.6(I)	0(R)	24.5 ± 0.4(S)	10.2 ± 0.2(R)	29.9 ± 0.6(S)	14.8 ± 0.2(I)	0(R)
	SMC302	25.4 ± 1.2(S)	20.4 ± 0.8(S)	15 ± 0.8(I)	9.6 ± 0.2(R)	9.5 ± 0.4(R)	15 ± 0.4(I)	0(R)	25 ± 0.8(S)	24.6 ± 0.6(S)	10 ± 0.8(R)
	SMC303	20 ± 0.4(S)	19.7 ± 0.4(S)	10 ± 0.4(R)	14.7 ± 0.4(I)	0(R)	24.7 ± 0.4(S)	24.9 ± 0.2(S)	20 ± 0.8(S)	10 ± 0.8(R)	0(R)
	SMC304	20 ± 0.8(S)	0(R)	0(R)	15 ± 0.4(I)	9.7 ± 0.4(R)	19.4 ± 0.6(S)	0(R)	20 ± 0.3(S)	20.2 ± 0.5(S)	16 ± 0.8(I)
	SMC305	26 ± 0.8(S)	23 ± 0.8(S)	0(R)	20.2 ± 0.6(S)	10.4 ± 0.2(R)	19.9 ± 0.6(S)	10.7 ± 0.6(R)	0(R)	19.8 ± 0.1(S)	15 ± 0.4(I)
	SMC306	15.4 ± 0.8(I)	9.9 ± 0.2(R)	10.5 ± 0.4(R)	12.5 ± 0.4(R)	12.5 ± 0.3(R)	10.5 ± 0.4(R)	8.5 ± 0.4(R)	10 ± 0.8(R)	10 ± 0.8(R)	0(R)
	SMC307	0(R)	0(R)	0(R)	12.9 ± 0.2(R)	0(R)	30 ± 0.4(S)	18.5 ± 0.3(I)	0(R)	0(R)	0(R)
	SMC308	0(R)	10.4 ± 0.8(R)	0(R)	11.7 ± 0.6(R)	0(R)	14.9 ± 0.6(I)	0(R)	0(R)	0(R)	0(R)

*R, resistant; I, intermediate; S, sensitive*.

### Antibacterial Activity

Antibacterial properties of all isolated endophytic bacteria were assessed against clinical strains of both Gram-negative and Gram-positive bacteria. The endophytic bacteria which inhibited the growth of any of the test organisms were considered to have antibacterial activity, and the zone of inhibition length was measured in mm (Table 6). Out of 33 endophytes screened, the majority showed antibacterial activity against *B. subtilis* followed by *K. pneumonia*. Bacterial strains, i.e., SMC204, SMC212, SMC101, and SMC301, exhibited antibacterial activity among all the six test organisms. The best activity was expressed by SMC212 against *S. aureus, B. subtilis, E. coli, S. typhi, K. pneumonia*, and *Pseudomonas aeruginosa* with a zone of inhibition having a diameter of 35, 30, 20, 15, 15, and 13 mm, respectively. Although several reports showed the antimicrobial potential of endophytic fungi from medicinal plants (Kuo et al., 2021), the antimicrobial evaluation of endophytic bacteria is rare (Cardoso et al., 2020). Li et al. (2008) investigated endophytic Actinomycetes associated with medicinal plants in the rainforest of Yunnan, China, and recognized that endophytic *Streptomyces* exhibit antimicrobial activities against *S. aureus, E. coli*, and *Candida albicans*.

**Table 6:** Antibacterial activity of isolated endophytic bacteria.

**Plant part**	**Bacterial code**	**Inhibition zone in mm**					
		**Test organisms**					
		***Bacillus subtilis***	***Escherichia coli***	***Staphylococcus aureus***	***Klebsiella pneumoniae***	***Salmonella typhi***	***Pseudomonas aeruginosa***
Root	SMC201	19.4 ± 1.6	2.2 ± 0.2	7 ± 0.8	–	–	–
	SMC202	15 ± 0.8	–	–	10.4 ± 0.4	–	–
	SMC203	17 ± 0.8	–	–	6.2 ± 0.6	–	–
	SMC204	15.4 ± 1.2	13.8 ± 0.6	12 ± 0.8	15.4 ± 1.2	15 ± 0.8	9.4 ± 0.8
	SMC205	11.4 ± 1.7	14.8 ± 0.2	4.7 ± 0.9	–	–	–
	SMC206	10 ± 2.4	–	–	–	–	–
	SMC207	16 ± 0.8	15 ± 0.8	34.7 ± 0.4	14 ± 0.8	9.9 ± 0.6	12 ± 0.8
	SMC208	–	10.8 ± 0.6	3.5 ± 0.4	–	–	9.5 ± 0.4
	SMC209	10 ± 1.6	–	4.7 ± 1.7	10 ± 0.4	–	–
	SMC210	–	5.5 ± 1.0	3 ± 0.8	12 ± 0.8	–	–
	SMC211	24.7 ± 1.2	4.7 ± 0.4	–	–	–	–
	SMC212	30 ± 0.8	20 ± 0.8	35 ± 0.8	15 ± 0.8	15 ± 0.8	13 ± 0.8
	SMC213	–	–	–	13.7 ± 0.6	6 ± 0.8	–
	SMC214	9.7 ± 2.05	3.4 ± 0.4	–	–	–	–
	SMC215	–	–	–	–	–	–
	SMC216	–	–	–	4 ± 0.8	–	–
Stem	SMC101	20 ± 1.6	12.2 ± 1.0	8.2 ± 1.0	3 ± 0.8	8 ± 0.8	10 ± 0.8
	SMC102	–	–	–	–	–	–
	SMC103	–	–	–	–	–	–
	SMC104	14 ± 0.8	10.2 ± 1.0	–	9.5 ± 0.4	5 ± 0.8	–
	SMC105	10.7 ± 1.6	–	2 ± 0.4	–	–	–
	SMC106	10.4 ± 1.2	–	4.2 ± 0.6	–	–	–
	SMC107	6 ± 0.8	–	5 ± 0.8	–	–	–
	SMC108	–	9.7 ± 0.4	–	6.2 ± 0.6	–	–
	SMC109	12.7 ± 1.7	–	–	–	–	–
Leaf	SMC301	14.3 ± 0.4	9.4 ± 0.8	8 ± 0.4	15 ± 0.3	12.2 ± 1.0	12 ± 0.8
	SMC302	19.7 ± 0.4	–	–	8 ± 0.8	3 ± 0.8	–
	SMC303	11 ± 0.8	–	10 ± 0.8	10 ± 0.8	–	–
	SMC304	11.8 ± 0.6	9.7 ± 0.4	11.9 ± 0.6	–	10 ± 0.4	15.4 ± 0.6
	SMC305	8.9 ± 0.6	10.5 ± 0.4	–	5 ± 1.1	–	–
	SMC306	–	–	–	–	–	–
	SMC307	20.4 ± 0.8	3 ± 0.8	9 ± 0.4	9.7 ± 0.4	5 ± 0.8	–
	SMC308	18 ± 0.8	6.4 ± 1.2	–	15 ± 0.8	10 ± 0.8	–
	Ampicillin	28 ± 0.8	26 ± 0.8	30 ± 1.6	27 ± 0.8	26 ± 0.8	25 ± 0.8
	Nutrient broth	–	–	–	–	–	–

### Molecular Identification of Endophytic Bacteria

**Figure 2:**
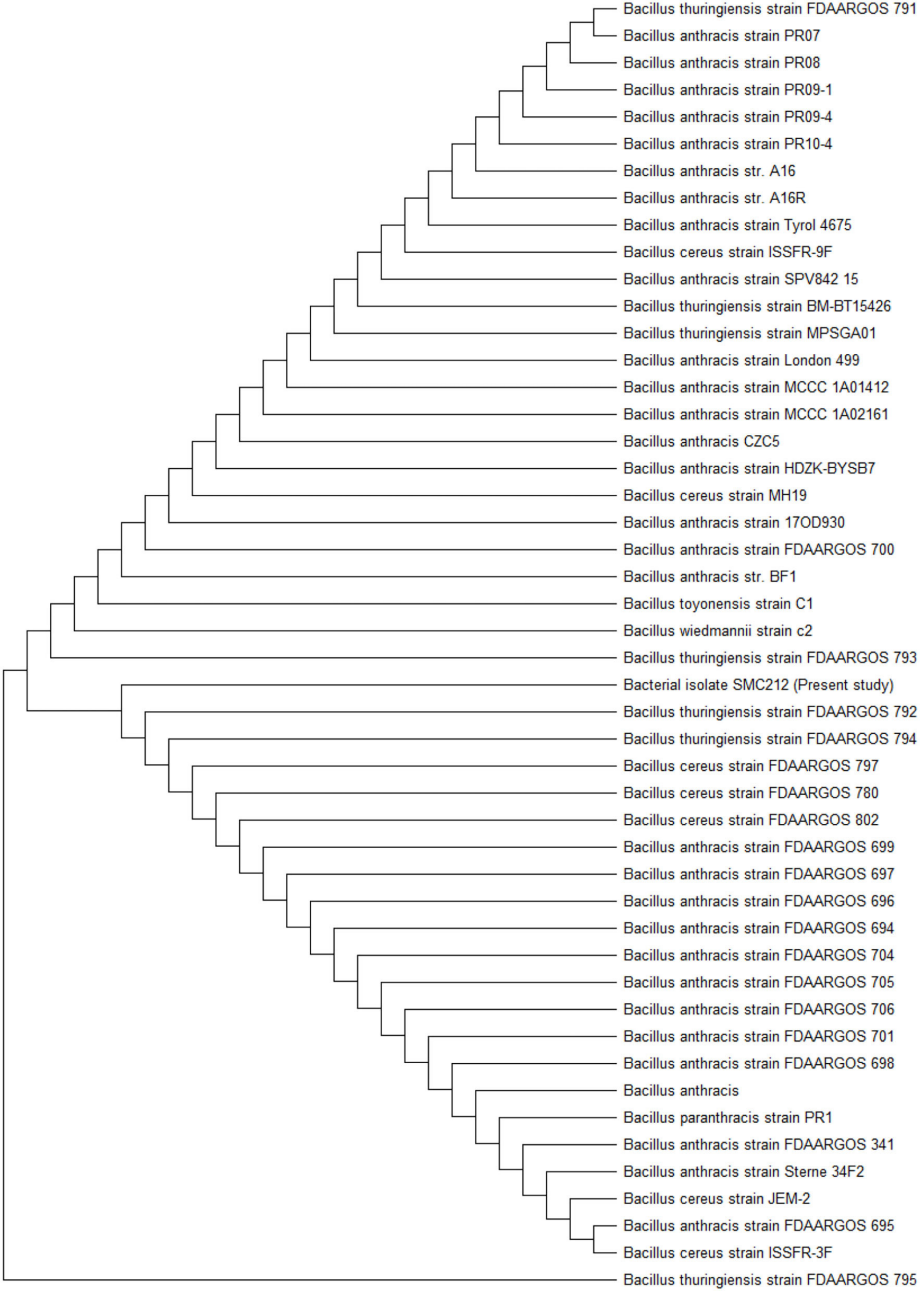
Maximum likelihood phylogenetic tree on the basis of 16S rDNA sequence of SMC212.

The 16S rRNA gene sequencing was used to characterize the endophytic bacterium SMC212, and a phylogenetic tree was constructed using MEGA 11 software. Using MEGA 11, the maximum likelihood tree of SMC 212, constructed based on 16s rDNA gene sequences analysis, was based on the Tamura 3-parameter model with the lowest BIC and highest AIC values. All spots with gaps and missing data were removed. Pairwise deletion was used to close gaps, and the estimated transition/transversion bias (R) was 2.2. The evolutionary history was inferred by using the maximum likelihood method and the bootstrap consensus tree inferred from 1,000 to 3,000 iterations. The evolutionary relationship is represented as a dendrogram (Figure 2), which clearly shows that SMC212 is related to *Bacillus thuringiensis*. To the best of our knowledge, this is the first article to report the isolation of an endophytic bacterium from *C. dichotoma* that has been identified and showed similarity to *B. thuringiensis*. The sequences obtained in this study have been deposited in GenBank under accession number OM320575. Numerous previous studies investigated the diversity of bacterial endophytes in medicinal plants (Hamayun et al., 2021). *Pseudomonas* sp., *Paenibacillus* sp., and *Bacillus megaterium* were previously identified as Korean ginseng root endophytes in the bacterial population isolated from *Plectranthus tenuiflorus* (Cho et al., 2007). *Paenibacillus* has been discovered as an endophyte in various woody plants such as coffee, pine, and poplar (Bent and Chanway, 2002).

### Chemical Constituents Using GC-MS Analysis

**Figure 3:**
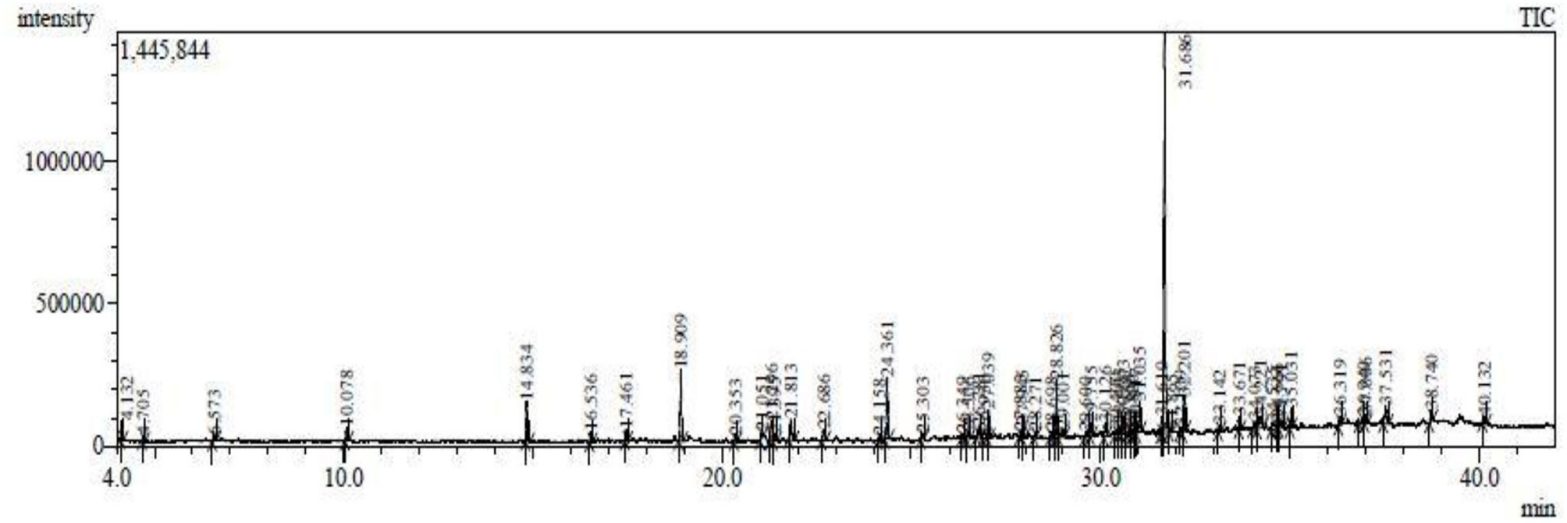
Abundance of the chemical compounds present in ethyl acetate extract of *Bacillus thuringiensis* SMC212.

The results pertaining to GC-MS analysis (Figure 3) of the ethyl acetate crude extract of *B. thuringiensis* OM320575 were analyzed using GC-MS which led to the identification of 38 different compounds. Table 7 shows the retention time (RT), molecular formula, and molecular weight. The NIST program was used to deconvolute the GC-MS data, and the obtained mass spectra were matched to entries in the compound library. The chemical structures of these compounds are also depicted in Figure 4 in the Supplementary Material. The compounds that occupied major percentage in the extract are dibutyl phthalate (32.53%) and eicosane (13.59%) at various time intervals; tetrapentacontane (6.88%) at various time intervals; heneicosane (6.58%) at various time intervals; hexadecane (5.57%); tetradecane (5.08%); silane, trichlorooctadecyl (2.6%); and 2,4-di-tert-butylphenol (2.27%). The bacterium *B. thuringiensis* OM320575 produced bioactive chemicals with antimicrobial, antioxidant, and anticancer properties, according to GC-MS analyses.

**Table 7:** Chemical composition of ethyl acetate extract of *Bacillus thuringiensis* SMC212 from GCMS analysis.

**S. No**.	**Retention time in minutes**	**Compounds**	**Molecular formula**	**Molecular weight g/mol**	**Peak area%**	**Activity**
1	4.13	Isobutyl acetate	C_6_H_12_O_2_	116	1.12	Antifungal (Xie et al., 2020)
2	4.70	Heptane 2,4dimethyl	C_9_H_20_	128	0.33	Biofuel (Strobel et al., 2010)
3	6.57	Benzene 1,3 dimethyl	C_8_H_10_	160	0.57	Antimicrobial (Abdelshafy Mohamad et al., 2020)
4	10.07	Decane	C_10_H_22_	142	0.9	Antibacterial (Xu et al., 2019)
5	14.83	Dodecane	C_12_H_26_	170	2.75	Antibacterial, Biofuel production (Kumaresan et al., 2015)
6	16.53	Dodecane 4,6 dimethyl	C_14_H_30_	198	0.75	Antibacterial (Li et al., 2021)
7	17.46	Sulfurous acid, nonyl 2 propyl ester	C_12_H_26_O_3_S	250	0.75	Antibacterial (Zaheer et al., 2021)
8	18.90	Tetradecane	C_14_H_30_	198	5.08	Antimicrobial (Dhouib et al., 2019)
9	20.35, 26.93	2,6,10 Trimethyl tridecane	C_16_H_34_	226	1.35	Antifungal (Zhang et al., 2015)
10	21.05	Nonane 5- butyl	C_13_H_28_	184	0.55	Antimicrobial (Munjal et al., 2016)
11	21.29, 22.68, 26.50, 27.03, 27.94, 30.49, 30.60, 33.67, 34.53, 35.03, 36.31	Eicosane	C_20_H_42_	282	13.59	Bronchodilators are drug used to treat throat problems (Alsultan et al., 2019)
12	21.39	Sulfurous acid, decyl 2-propyl ester	C_15_H_32_O_3_S	292	0.9	Antibacterial (Zaheer et al., 2021)
13	21.81	2,4-Di-tert-butylphenol	C_14_H_22_O	206	2.27	Antibacterial (Mishra et al., 2020)
14	24.15	1-Heptadecene	C_17_H_34_	238	0.46	Antimicrobial (Devi and Singh, 2013)
15	24.36	Hexadecane	C_16_H_34_	226	5.57	Plant metabolite (Phillips et al., 2008)
16	25.30	5,5- Diethyltridecane	C_17_H_36_	240	0.49	–
17	26.34, 26.78	Heptadecane	C_17_H_36_	240	1.64	Antioxidant, Antiproliferative, Antimutagenic (Kaur et al., 2020)
18	27.88	Octadecane,5- methyl-	C_19_H_40_	268	0.34	Antibacterial (Nascimento et al., 2012)
19	28.27	Heptadecane, 3- methyl	C_18_H_38_	254	0.31	Antifungal (Gao et al., 2017)
20	28.69	n-Hetadecanol-1	C_17_H_36_O	256	0.32	–
21	28.82, 32.20	Heneicosane	C_21_H_44_	296	6.58	Antibacterial, Antitumor, oviposition-inducing pheromone [for trapping mosquitoes (Abdel-Hady et al., 2016)]
22	29.00	Heptacosane, 1-chloro-	C_27_H_55_Cl	415	1.23	Antibacterial, Anti-inflammatory (Abdel-Hady et al., 2016)
23	29.6	11- Methyltricosane	C_24_H_50_	338	0.86	–
24	29.75	Octane,2,6,6-trimethyl-	C_11_H_24_	156	1.08	Antifungal (Wang et al., 2013)
25	30.12	1,2-Benedicarboxylic acid, bis(2-methylpropyl) ester	C_16_H_22_O_4_	278	1.14	–
26	30.4	Pentadecane, 4-methyl	C_16_H_34_	226	0.74	Antimicrobial (Tapfuma et al., 2020)
27	30.76, 34.07, 34.22, 34.79, 37.53, 38.74	Tetrapentacontane	C_54_H_110_	759	6.88	Antibacterial (Dhankhar et al., 2013)
28	30.836	5,5- Diiethylpentadecane	C_19_H_40_	268	0.39	–
29	30.91, 33.14	Tetracosane	C_24_H_50_	338	1.04	Antimicrobial (Abdelshafy Mohamad et al., 2020)
30	31.03	Silane, trichlorooctadecyl-	C_18_H_37_Cl_3_Si	387	2.6	–
31	31.61	1-(+)- Ascorbic acid 2,6-dihexadecanoate	C_38_H_68_O_8_	652	1.37	Antimicrobial, Antioxidant (Khan et al., 2020; Radhakrishnan and Mathew, 2020)
32	31.68	Dibutyl phthalate	C_16_H_22_O_4_	278	32.53	Antimicrobial (Aboobaker et al., 2019)
33	31.86	Diglycolic acid, 2-ethylbutyl propyl ester	C_13_H_24_O_5_	260	0.34	–
34	32.10	1-Tetracosene	C_24_H_48_	336	0.36	Antibacterial (Tyagi and Singh, 2020)
35	34.66	2-Methylhexacosane	C_27_H_56_	380	0.82	Anticancerous (Salim, 2018)
36	36.94	Hexacontane	C_60_H_122_	843	0.7	Antibacterial (Sheoran et al., 2015)
37	37.00	6-Bromohexanoic acid, 4-hexadecyl ester	C_22_H_43_BrO_2_	419	0.68	–
38	40.13	Dotriacontane	C_32_H_66_	450	0.6	Antioxidant (Koksal et al., 2011)

## Discussion

In this study, a total of 33 bacterial strains were recovered from plant segments collected from selected *C. dichotoma* plant parts such as root, stem, and leaf collected from SMVDU, Katra, Jammu, for the isolation of endophytic bacteria. In India, a countable number of reports showed the diversity of endophytic bacteria and fungi in medicinal plants (Praptiwi et al., 2020), but to our best of knowledge, this is the first-ever report on endophytic bacteria from *C. dichotoma*, particularly in Jammu region. In this study, the surface sterilization method was optimized to get maximum endophytic bacteria from the medicinal plant *C. dichotoma*. The population density of epiphytes or rhizospheric bacteria is more as compared with endophytic bacteria. To avoid contamination for the isolation of endophytic bacteria, plant samples must be properly surface sterilized before inoculating them into the nutrient agar medium. The simple and powerful method of surface sterilization was applied for the isolation of endophytic bacteria from the root, stem, and leaf tissues. The study found that surface sterilization of *C. dichotoma* medicinal plant for the isolation of endophytic bacteria using 70% ethanol was not effective to remove contamination on the plant surface. Hence, it was evaluated that 70% ethanol was not efficient in removing epiphytic bacteria. Although mercuric chloride was found to be a good decontaminating agent, surface sterilization of *C. dichotoma* using mercuric chloride was not found effective because the survival percentage of endophyte decreased. In our study, a high concentration of (4%) sodium hypochlorite was found to be more effective than the low concentration of (1%) in removing plant surface microorganisms. Hence, sodium hypochlorite showed very low contamination because it is very effective as a disinfectant agent against many contaminated bacteria as previously reported (Khanam and Chandra, 2017). Hypochlorite acts as a strong oxidant that can denature by aggregating necessary proteins of bacteria as reported earlier (Winter et al., 2008). Our results are in acceptance with previous studies on attempts using several sterilization methods (Daud et al., 2012). In brief, a combination of sodium hypochlorite, ethanol, and Tween 20 was found to be very effective in removing epiphytic microorganisms. The earlier study used 70% alcohol for 2 min, 2% sodium hypochlorite for 3 min, and sterile distilled water for 2 min for surface sterilization (Cardoso et al., 2020).

Another part of the study was a preliminary characterization of isolated endophytic bacteria. Endophytic bacteria exhibited a broad range of morphological and biochemical characteristics which designated that they are different bacterial species. The endophytic bacteria multiply and inhabit in the plants where the environment carries high ionic strength. Earlier, many studies reported that the endophytic bacteria tolerate the high salt concentration (Kumar et al., 2015). In this study, the bacterial endophytes were able to grow differentially at different salt concentrations. In this study, 22 of 33 bacterial isolates exhibited sustained growth at a NaCl concentration of 12%. The earlier study found that *Bacillus* sp. tolerated up to 2% NaCl, while *Pseudomonas* sp. 4% NaCl (Rashid et al., 2012). The endophytic bacterial isolates of *Momordica charentia* exhibited tolerance to NaCl concentration of 4–10% (Singh et al., 2013).

In plants, endophytes are chemical synthesizers. They are capable of producing bioactive compounds that can be used by plants for defense against pathogens, and some of these products have been proved for useful drug discovery (Bungtongdee et al., 2019). To date, most of the natural compounds from endophytes are used as anticancerous, antibacterial, antifungal, antiviral, antidiabetic, and other bioactive products because of their different functional roles (Guo et al., 2008). In this study, out of 33 isolates, 4 showed antibacterial activity against all the six test organisms, i.e., *B. subtilis, E. coli, K. pneumonia, S. aureus, S. typhi*, and *P. aeruginosa*, which is similar to an earlier report (Sun et al., 2013). Endophytic bacterial variety was found to be abundant in *C. dichotoma*. *Bacillus* sp., a Gram-positive bacterium, was found as endophytic with considerable antibacterial activity. *Bacillus* sp. was shown to be the closest homolog to *B. thuringiensis*, an endophytic *Bacillus*. In many instances, isolates belonging to this genus have been found to produce antimicrobial and other bioactive compounds (Hateet, 2020). According to Beiranvand et al. (2017), endophytic *B. thuringiensis* isolated from Iranian medicinal plants produced a broad range of antimicrobial compounds. Similarly, Islam et al. (2019) discovered that *B. thuringiensis* isolated from several gymnosperms and angiosperms has antibacterial activity. Endophytic bacteria found in plant tissues could be a new source of bioactive compounds, according to these studies.

The appearance of antibiotic resistance among pathogenic microorganisms restricts treatment alternatives (Mengoni et al., 2014). In addition to clinical pathogens, antibiotic-resistant genes are also present in environmental strains that are horizontally transferred to other microorganisms (Christina et al., 2013). In this study, susceptibility pattern of isolated endophytic bacteria was studied using 10 different antibiotics. Most of the isolates were resistant to rifampicin, amoxicillin, and bacitracin, while they were susceptible to erythromycin and streptomycin. Kumar et al. (2015) found that the bacterial strain *Pseudomonas* sp. from *Cassia tora* was resistant to chloramphenicol and amoxicillin.

The substances found in *B. thuringiensis* OM320575 crude extracts are predominantly alcohols, terpenes, alkaloids, hydrocarbons, and their derivatives. These chemicals are renowned for their therapeutic effects and have been found in endophytes isolated from medicinal plants (Tapfuma et al., 2020). Some of these chemicals are separated and utilized as antimicrobials individually in extracts. The principal chemical components found in the *B. thuringiensis* OM320575 ethyl acetate extract include dibutyl phthalate, eicosane, tetrapentacontane, heneicosane, hexadecane, tetradecane, silane, trichlorooctadecyl, and 2,4-di-tert-butylphenol. Dibutyl phthalate is one of the most significant chemicals found in the strain SMC 212, and it may play a role in microbial inhibition. Similarly, Wilkins et al. (2000) reported that *Trichoderma viride* produced pathogen-inhibiting volatile metabolites such as 2-propanol, 3-methylfuran, methyl-1-propanol, 1-pentanol, and 2-hexanone. Additionally, pentanones, octanones, non-anones, and undecanones have been described in *T. atroviride* culture (Nemčovič et al., 2008) and heptanone by *T. viride* (Siddiquee et al., 2012). Sulfurous acid, heptadecane, and octane identified in this investigation have fumigant, insecticidal, and fungicidal activities. Of these, octadecane, heptadecane, and di-tert-butylphenol are known to be emitted by plants under stress. The antifungal activity of eicosane (C_20_H_42_) and dibutyl phthalate (C_16_H_22_O_4_) was discovered in larger percentages in the ethyl acetate fraction of *Streptomyces* strain (Ahsan et al., 2017).

## Conclusion

This may be the first-ever report on endophytic bacteria isolated from *C. dichotoma* in Jammu region, and our findings indicate the high diversity of endophytic bacterial strains associated with the root, stem, and leaf of the medicinal plant that differed appreciably in their morphological, physiological, and biochemical features. This study revealed that *C. dichotoma* is a potential but underexploited resource for bioactive bacterial endophytes since the exploited bacteria isolated from *C. dichotoma* showed promising results for antimicrobial and enzymatic activities, utilization of various carbon sources, and tolerance for high salt concentration (12% NaCl). Antimicrobial estimation revealed that bacterial endophytes showed significant antibacterial activity against *S. typhi, E. coli, P. aeruginosa, B. subtilis, S. aureus*, and *K pneumonia*. The therapeutic properties of *C. dichotoma* may be a consequence of its endophytic microorganisms producing biologically active products. The bacterial strains were sensitive to antibiotic erythromycin and streptomycin, whereas most of them were resistant to rifampicin, amoxicillin, and bacitracin. Endophytes have been found to be abundant sources of novel natural chemicals with a wide range of biological functions and a high level of structural diversity. By synthesizing dibutyl phthalate; eicosane; tetrapentacontane; heneicosane; hexadecane; tetradecane; silane, trichlorooctadecyl; and 2,4-di-tert-butylphenol as bioactive chemicals, one potential endophyte isolated from *C. dichotoma* and identified as *B. thuringiensis* OM320575 by 16S rRNA demonstrated considerable antibacterial activity against pathogenic bacteria. Further exploration would provide us an insight into the potential use of isolated bacterial endophytes, and it will lead to the discovery of various high-value metabolites.

## Data Availability Statement

The original contributions presented in the study are included in the article/Supplementary Material, further inquiries can be directed to the corresponding author/s.

## Author Contributions

MS conducted the experiments, recorded the observations, analyzed the results, and prepared the draft manuscript. SM contributed to designing the experiments, providing the facilities, analyzing the results, and edited the manuscript. All authors contributed to the article and approved the submitted version.

## Conflict of Interest

The authors declare that the research was conducted in the absence of any commercial or financial relationships that could be construed as a potential conflictof interest.

## Publisher's Note

All claims expressed in this article are solely those of the authors and do not necessarily represent those of their affiliated organizations, or those of the publisher, the editors and the reviewers. Any product that may be evaluated in this article, or claim that may be made by its manufacturer, is not guaranteed or endorsed by the publisher.
