# Supplementary material for: Diversity, Antimicrobial Activity, and Antibiotic Susceptibility Pattern of Endophytic Bacteria Sourced From Cordia dichotoma L
Source: Front Microbiol. 2022 May 13;13:879386. doi: 10.3389/fmicb.2022.879386 (PMC9136406; doi:10.3389/fmicb.2022.879386)
Supplement: Supplementary file 1 [file Data_Sheet_1.docx]

**Supplementary material**

**Table S1 Composition of culture media for enzyme activity (For 100 ml)**

| **Cellulase** | **Amylase** | **Lipase** |
| --- | --- | --- |
| CMC : 1 g | Yeast extract : 3 g | Yeast extract : 0.3 g |
| Yeast extract : 0.4 g | Strach : 1 g | Peptone : 0.5 g |
| Peptone : 0.4 g | Agar : 1.2 g | Agar : 1.5 g |
| NaCl: 0.25 g | NaCl : 0.5 g | Tributyrin : 1 ml |
| MgSO_4_ : 0.02 g |  |  |
| KH_2_PO_4_ : 0.1 g |  |  |
| Agar : 2 g |  |  |


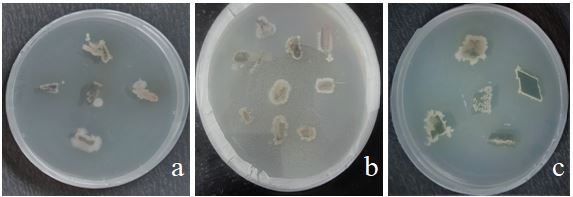


**Fig. S2 a, b, c indicates growth of bacterial endophyte from root, stem and leaf segments respectively of *C. dichotoma.***

**Table S3 Cultural characterization of the isolated endophytic bacteria**

| **Plant part** | **Bacterial code** | **Colour** | **Size** | **Margin** | **Texture** | **Consistency** |
| --- | --- | --- | --- | --- | --- | --- |
| **Root** | SMC201 | White | Small | Regular | Raised | Creamy |
|  | SMC202 | Milky white | Small | Circular | Raised | Creamy |
|  | SMC203 | Colourless | Small | Irregular | Raised | Creamy |
|  | SMC204 | Colourless | Large | Irregular | Flat | Slimy |
|  | SMC205 | Colourless | Large | Irregular | Raised | Creamy |
|  | SMC206 | Orange | Small | Irregular | Raised | Mucoid |
|  | SMC207 | Colourless | Small | Circular | Raised | Slimy |
|  | SMC208 | Orange | Small | Irregular | Raised | Creamy |
|  | SMC209 | Yellow | Small | Irregular | Flat | Creamy |
|  | SMC210 | White | Small | Irregular | Flat | Creamy |
|  | SMC211 | Greenish yellow | Large | Circular | Raised | Creamy |
|  | SMC212 | Colourless | Small | Irregular | Raised | Slimy |
|  | SMC213 | Colourless | Small | Regular | Raised | Slimy |
|  | SMC214 | Milky White | Small | Circular | Flat | Slimy |
|  | SMC215 | Yellow | Small | Irregular | Raised | Creamy |
|  | SMC216 | Colourless | Small | Irregular | Raised | Mucoid |
| **Stem** | SMC101 | White | Small | Irregular | Flat | Slimy |
|  | SMC102 | Orange | Large | Irregular | Raised | Creamy |
|  | SMC103 | Milky white | Large | Circular | Raised | Creamy |
|  | SMC104 | Colourless | Small | Circular | Flat | Creamy |
|  | SMC105 | Yellow | Small | Irregular | Raised | Slimy |
|  | SMC106 | Colourless | Small | Regular | Raised | Creamy |
|  | SMC107 | Colourless | Small | Irregular | Raised | Creamy |
|  | SMC108 | Milky white | Small | Irregular | Raised | Mucoid |
|  | SMC109 | Colourless | Large | Irregular | Flat | Creamy |
| **Leaf** | SMC301 | Milky white | Small | Circular | Raised | Creamy |
|  | SMC302 | Colourless | Small | Circular | Raised | Creamy |
|  | SMC303 | Colourless | Small | Irregular | Raised | Slimy |
|  | SMC304 | White | Large | Irregular | Flat | Slimy |
|  | SMC305 | Colourless | Small | Regular | Raised | Creamy |
|  | SMC306 | Orange | Small | Irregular | Raised | Creamy |
|  | SMC307 | Milky white | Small | Irregular | Flat | Slimy |
|  | SMC308 | Colourless | Small | Irregular | Raised | Creamy |
|  |  |  |  |  |  |  |

**
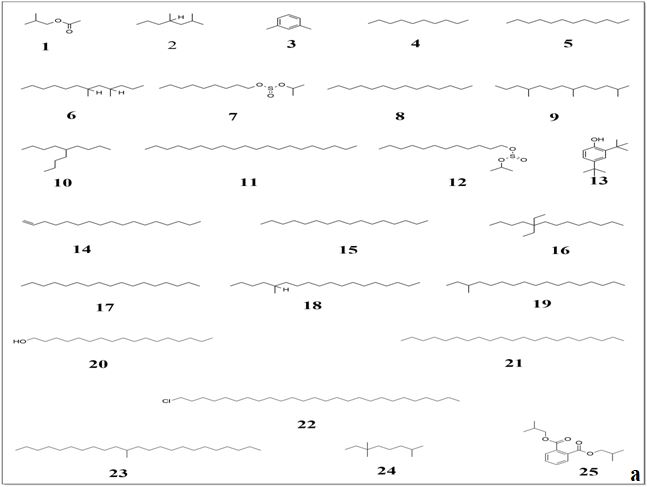

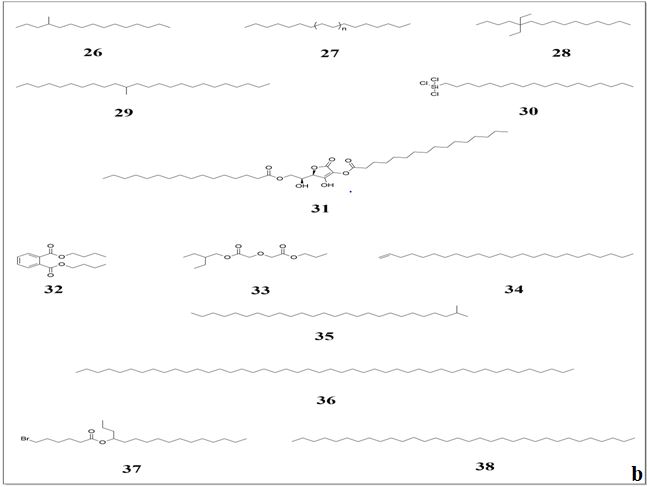
**

**Fig. S4 a, b indicates chemical structures of thirty eight different compounds identified from ethyl acetate fraction of *Bacillus thuringiensis* OM320575 by GC-MS analysis as mentioned in Table 9**
